# Supplementary material for: Metabolite and Transcriptome Profiles of Proanthocyanidin Biosynthesis in the Development of Litchi Fruit
Source: Int J Mol Sci. 2022 Dec 28;24(1):532. doi: 10.3390/ijms24010532 (PMC9820520; doi:10.3390/ijms24010532)
Supplement: Supplementary file 1 [file ijms-24-00532-s001.zip › ijms-2067386-supplementary.pdf]

International Journal of
Molecular Sciences

MDPI

Table S1. qRT-PCR specific primer sequences of litchi *ANR*, *LAR* and *ANS* genes.

qRT-PCR prime	Primer sequence (5'-3')
Actin_F	ACCGTATGAGCAAGGAAATCACTG
Actin_R	TCGTCGTACTCACCCTTTGAAATC
ANR1a_F	CGCCACGTCCTTGATTACAG
ANR1a_R	GGAACTTAGCAGCCTCAGGA
ANR1b_F	GTTTTCCATACTGCCTCGCC
ANR1b_R	TCCACAGAACAGCAGCCATA
ANR2a_F	TGCCAATTACTGCCAGCAAA
ANR2a_R	AGATCACCATGCCTCCTAGC
ANR2b_F	AGTTGGAGACCGTTACCCTG
ANR2b_R	GCACACAAGAATCTGCCACA
ANR3_F	TGGTTGGTACTTCTTCGGCT
ANR3_R	CTCCGCATGTACAATGGCAA
LAR1_F	CCATTTTCGACCACCAAGCA
LAR1_R	GTGAATGATGCCACGATGCT
LAR2_F	GTACAGGGAAAAGCGCAGAG
LAR2_R	GTATGCTTTGACGGTGCCAT
ANS1_F	CCTTCCACGTCATCAACCAC
ANS1_R	CGAAGTAGTCCCTCCAGTCC
ANS2_F	CCTTCCACGTCATCAACCAC
ANS2_R	CGAAGTAGTCCCTCCAGTCC

Table S2. Summary information of sequencing data and mapped ratios.

Samples	Total Reads	GC Content	% ≥ Q30	Mapped Reads
30 DAF-1	41,382,120	46.48%	94.39%	36,851,805 (89.05%)
30 DAF-2	37,247,892	48.64%	94.01%	33,618,260 (90.26%)
30 DAF-3	43,427,130	46.67%	94.17%	38,883,008 (89.54%)
60 DAF-1	35,550,750	48.04%	94.58%	31,806,580 (89.47%)
60 DAF-2	37,725,280	47.34%	93.79%	33,535,383 (88.89%)
60 DAF-3	41,255,920	46.83%	94.06%	36,643,570 (88.82%)
75 DAF-1	37,649,738	46.64%	94.09%	33,621,624 (89.30%)
75 DAF-2	38,195,216	46.57%	94.06%	34,058,772 (89.17%)
75 DAF-3	42,363,658	46.38%	94.21%	38,030,817 (89.77%)
85 DAF-1	37,193,802	46.57%	94.70%	33,157,788 (89.15%)
85 DAF-2	37,151,122	46.78%	94.08%	33,078,054 (89.04%)
85 DAF-3	37,785,300	46.43%	94.34%	33,515,026 (88.70%)

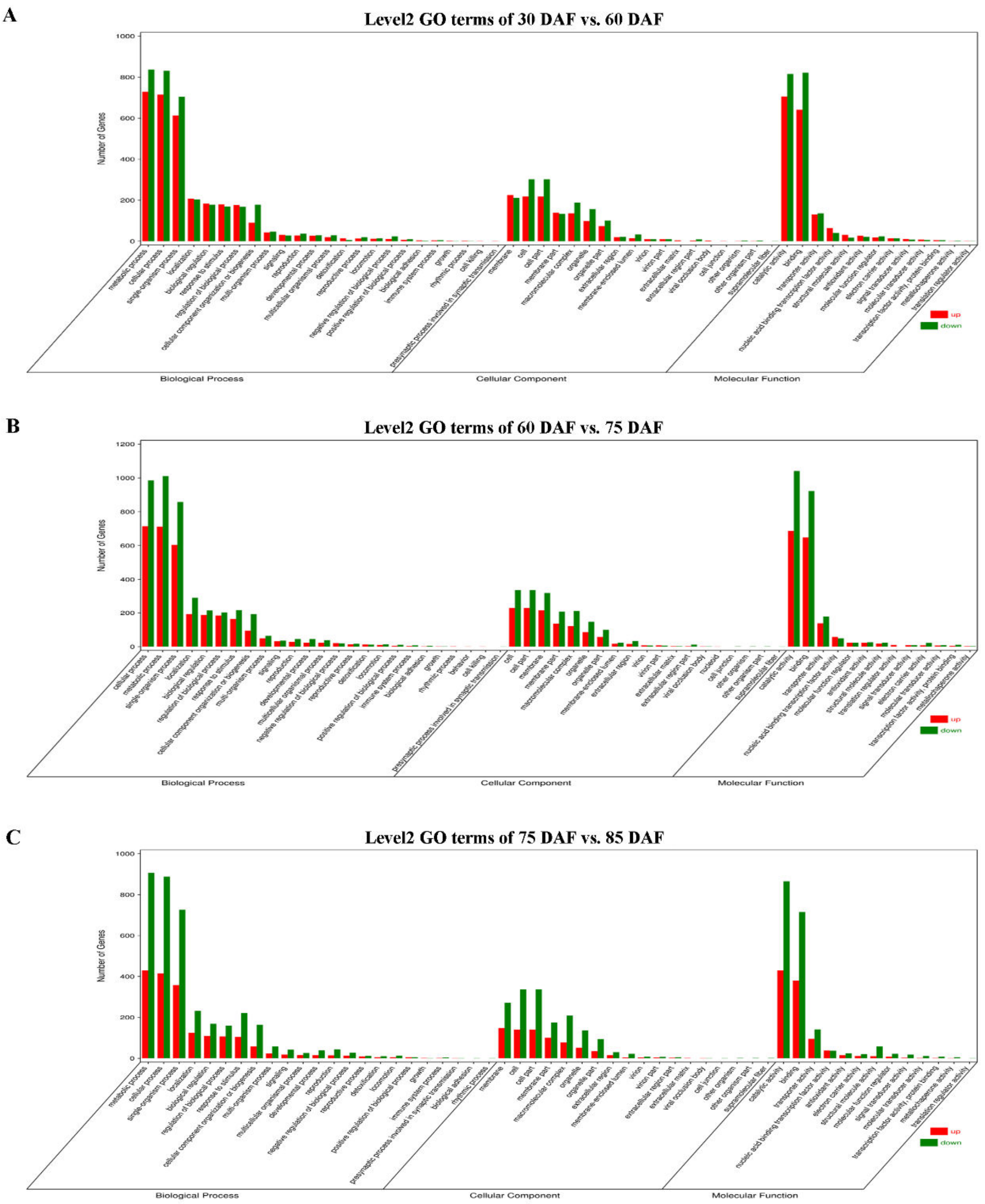

Figure S1. GO function of DEGs identified during litchi fruit development. (A), (B), and (C) represent the GO functions of DEGs at 30 DAF vs. 60 DAF, 60 DAF vs. 75 DAF, and 75 DAF vs. 85 DAF, respectively.

Table S3. Accession numbers of the sequences from various plant species used in the phylogenetic trees of ANR, LAR and ANS proteins

Gene Name	Gene Number	Latin Name
MiANR1/2/3	MG322128/MG322129/MG322130	*Mangifera indica*
CsANR1/2	KY615701/KY615702	*Camellia sinensis*
GmANR1/2	NM_001254984/NM_001256143.1	*Glycine max*
MdANR1/2	JN035299/JN035300	*Malus domestica*
HvANR1/2	AK373696/AK365124	*Hordeum vulgare*

VvANR	DQ129684	*Vitis vinifera*
AtANR	NM_104854	*Arabidopsis thaliana*
MtANR	AAN77735	*Medicago truncatula*
MdLAR1/2	DQ139836/DQ139837	*Malus domestica*
PuLAR1/2	MW928480/MW928481	*Populus ussuriensis*
VvLAR1/2	DQ129686/DQ13983	*Vitis vinifera*
PcLAR1/2	BN000698/BN000164	*Phaseoluscoccineus*
GrLAR1/2	BN000700/BN000701	*Gossypium raimondii*
CsLARa/b	KY615698/KY615700	*Camellia sinensis*
TcANS	ADD51356	*Theobroma cacao*
PtANS1/2	XP_002298081/XM_002304416	*Populus trichocarpa*
VvANS	EU156063	*Vitis vinifera*
GmANS1/2	KC493686/AY382829	*Glycine max*
CsANS1/2	XP_028072725/XP_028087321	*Camellia sinensis*
MdANS	AF117269	*Malus domestica*
NtANS1/2	NP_001312972/NP_001312183	*Nicotiana tabacum*
TaANS	BAE98276	*Triticum aestivum*

Table S4. Accession numbers of the MYB sequences from various plant species used in phylogenetic tree of MYB transcription factors in Figure 7C.

Gene Name	Gene Number	Latin Name
LcMYBPA1/2	LITCHI017552/LITCHI019503	*Litchi chinensis*
DkMYB2/4	AB503699/AB503701	*Diospyros kaki*
VvMYBPA1/2	AM259485/EU919682	*Vitis vinifera*
AtTT2/AtPAP1	CAC40021/AAG42001	*Arabidopsis thaliana*
MdMYB1/10	ABK58136/DQ267896	*Malus domestica*
SlANT1/SlAN2	AAQ55181/FJ705320	*Lycopersicon esculentum*

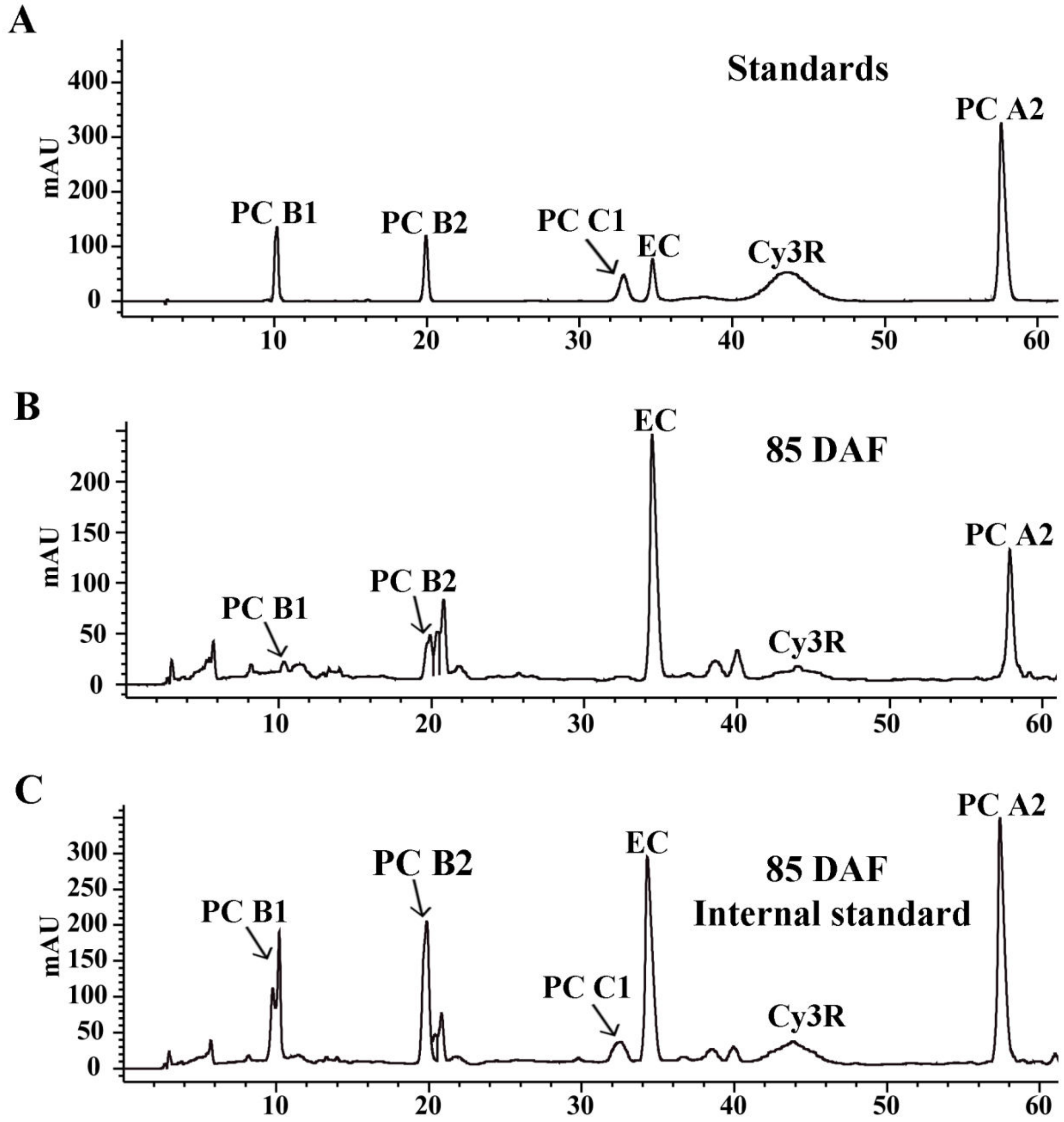

Figure S2. HPLC internal standard method for identification the PA substances in the soluble PA extract of litchi pericarp at 85 DAF. (A) HPLC chromatograms of mixed standards of a variety of (epi)catechin/PCs. (B) HPLC chromatograms of soluble PA extract of litchi pericarp samples at 85 DAF. (C) HPLC chromatograms of the extract in (B) with addition of mixed standards. Addition of the amount of 0.3 mg/mL of EC and PC A2/ B2, and 0.75 mg/mL of Cy3R led to increase in the respective peaks of the identified substances.

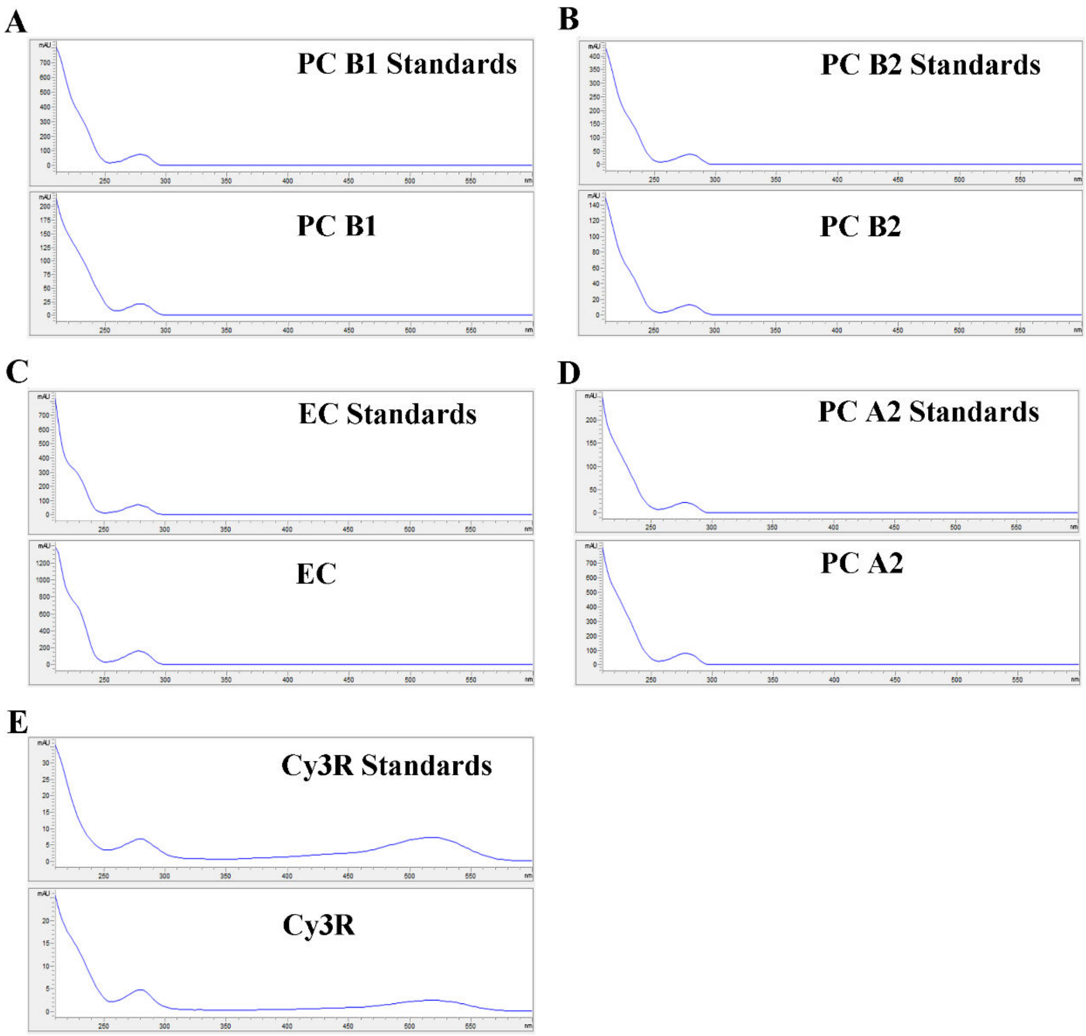

Figure S3. Comparison of the full absorption spectra (190- 600 nm) of the identified PAs and anthocyanin in the extracts from litchi pericarp with those of the standards. (A), (B), (C), (D) and (E), represent the full absorption spectra detected in the extract and standards of PC B1, PC B2, EC, PC A2, Cy3R , respectively.

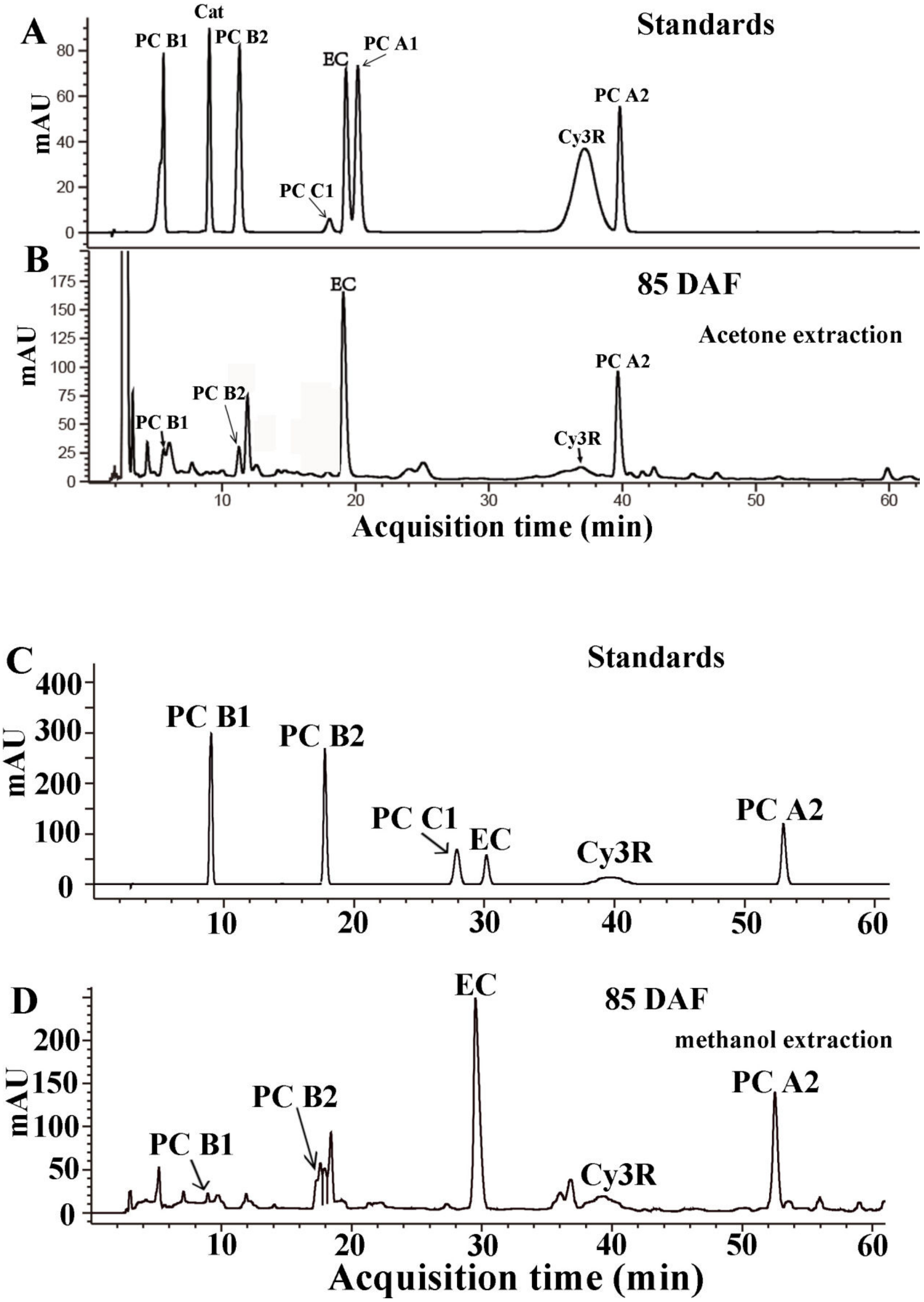

Figure S4. Comparison of acetone and methanol extraction for the soluble PA extract of litchi pericarp at 85 DAF. HPLC chromatograms of same mixed standards of a variety of (epi)catechin/PCs in different HPLC separation method (A, C). HPLC chromatograms of soluble PA extract of litchi pericarp samples at 85 DAF by acetone (B) and methanol extraction (D). Cat in (A) is a symbol of (+)-catechin.

A

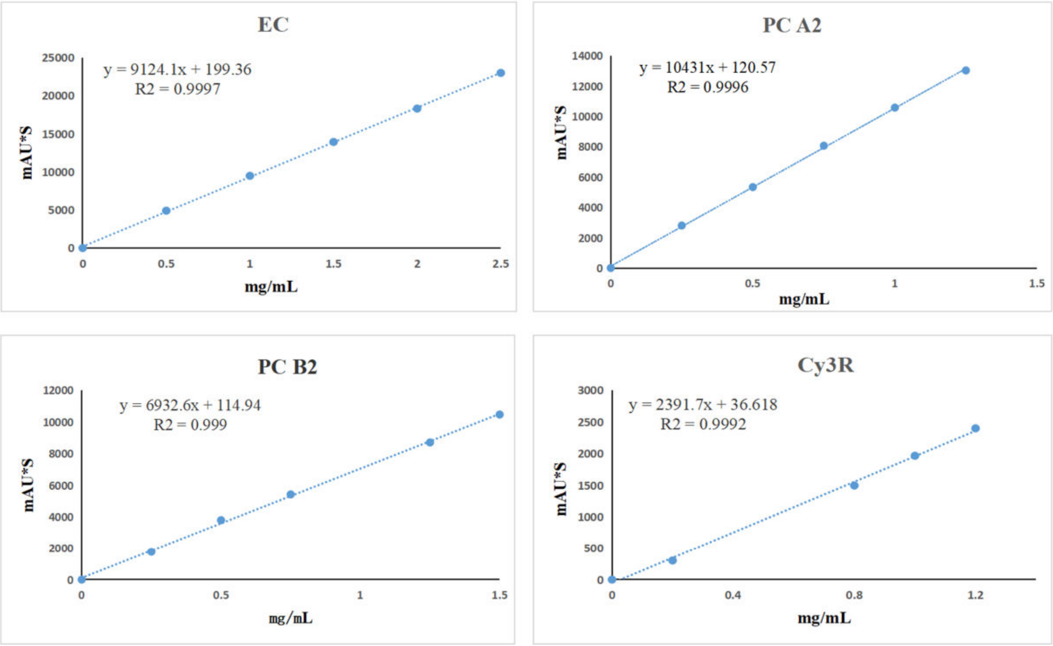

B

Sample \ Average mAU*s	EC	PC A2	PC B2	Cy3R
30 DAF	30741.43333	17087.8	3235.085773	0
60 DAF	13416.63333	8403.06608	2081.58423	0
75 DAF	9298.982097	6257.76253	1507.03284	881.31134
85 DAF	8334.674317	4573.848633	1936.134767	2373.8667

Figure S5. The standard curves of individual PAs and Cy3R (A) and chromatographic peak area of the identified substances of litchi pericarp samples at the four stages (B).
